# Parvifloron D-based potential therapy for glioblastoma: Inducing apoptosis *via* the mitochondria dependent pathway

**DOI:** 10.3389/fphar.2022.1006832

**Published:** 2022-10-12

**Authors:** Mariana Magalhães, Eva María Domínguez-Martín, Joana Jorge, Ana Cristina Gonçalves, Ana María Díaz-Lanza, Bruno Manadas, Thomas Efferth, Patrícia Rijo, Célia Cabral

**Affiliations:** ^1^ PhD Programme in Experimental Biology and Biomedicine, Institute for Interdisciplinary Research (IIIUC), University of Coimbra, Coimbra, Portugal; ^2^ CNC—Center for Neuroscience and Cell Biology, University of Coimbra, Coimbra, Portugal; ^3^ Coimbra Institute for Clinical and Biomedical Research (iCBR), Clinic Academic Center of Coimbra (CACC), Faculty of Medicine, University of Coimbra, Coimbra, Portugal; ^4^ Center for Innovative Biomedicine and Biotechnology (CIBB), University of Coimbra, Coimbra, Portugal; ^5^ CBIOS—Universidade Lusófona’s Research Center for Biosciences & Health Technologies, Lisbon, Portugal; ^6^ Departamento de Ciencias Biomédicas, Facultad de Farmacia, Universidad de Alcalá de Henares, Madrid, Spain; ^7^ Laboratory of Oncobiology and Hematology, University Clinic of Hematology and Applied Molecular Biology, Faculty of Medicine, University of Coimbra, Coimbra, Portugal; ^8^ iCBR, Group of Environment Genetics and Oncobiology (CIMAGO)—Faculty of Medicine, University of Coimbra, Coimbra, Portugal; ^9^ Department of Pharmaceutical Biology, Institute of Pharmaceutical and Biomedical Sciences, Johannes Gutenberg University, Mainz, Germany; ^10^ Faculty of Pharmacy, Instituto de Investigação do Medicamento (iMed.ULisboa), University of Lisbon, Lisbon, Portugal; ^11^ Centre for Functional Ecology, Department of Life Sciences, University of Coimbra, Coimbra, Portugal

**Keywords:** glioblastoma, *Plectranthus* spp., abietane diterpenes, antitumor activity, molecular mechanisms

## Abstract

Glioblastoma (GB) is the most malignant and frequent primary tumor of the central nervous system. The lack of diagnostic tools and the poor prognosis associated with this tumor type leads to restricted and limited options of treatment, namely surgical resection and radio-chemotherapy. However, despite these treatments, in almost all cases, patients experience relapse, leading to survival rates shorter than 5 years (∼15–18 months after diagnosis). Novel therapeutic approaches are urgently required (either by discovering new medicines or by repurposing drugs) to surpass the limitations of conventional treatments and improve patients’ survival rate and quality of life. In the present work, we investigated the antitumor potential of parvifloron D (ParvD), a drug lead of natural origin, in a GB cell line panel. This natural drug lead induced G2/M cell cycle arrest and apoptosis *via* activation of the intrinsic mitochondria-dependent pathway. Moreover, the necessary doses of ParvD to induce pronounced inhibitory effects were substantially lower than that of temozolomide (TMZ, first-line treatment) required to promote comparable effects. Therefore, ParvD may have the potential to overcome the resistance related to TMZ and contribute to the pursuit of hopeful treatments based on ParvD as a drug lead for future chemotherapeutics.

## Introduction

Glioblastoma (GB) is the most common and malignant primary glioma of the central nervous system (CNS), including brain and spinal cord tumors (Louis et al., 2021). According to the World Health Organization (WHO), GB is classified as a grade IV adult-type diffuse glioma that comprises the *isocitrate dehydrogenase* (*IDH*)-wildtype tumors with shared genetic mutations (Brat et al., 2018; Tesileanu et al., 2020; Louis et al., 2021; Wen and Packer, 2021; WHO Classification of Tumours Editorial Board, 2021). Moreover, GB could arise *de novo* or following lower-grade tumors (Louis et al., 2016). Recent data from the Global Cancer Observatory (GLOBOCAN) and WHO revealed that brain and CNS tumors are more prevalent in older individuals (approx. 62 years of age) and affect more males than females (Louis et al., 2016; International Association of Cancer Registries (IARC), 2020; Wen and Packer, 2021).

In spite of the efforts made to increase patient survival, GB still has low survival rates (∼15–18 months after diagnosis), which is mainly attributed to the late diagnosis of this tumor (Alifieris and Trafalis, 2015; Wick et al., 2018; Wen et al., 2021). Surgical resection followed by concomitant chemoradiotherapy and adjuvant chemotherapy (temozolomide (TMZ)) is the standard of care for GB patients (Alifieris and Trafalis, 2015; Wick et al., 2018; Le Rhun et al., 2019; Wen et al., 2021). However, these treatments have several limitations, such as the incomplete resection of the tumor and the development of multidrug resistance (MDR) associated with TMZ therapy that contribute to tumor relapse (Alifieris and Trafalis, 2015; Fatai and Gamieldien, 2018; Wick et al., 2018; Le Rhun et al., 2019). Therefore, the continuous search to find more efficient and targeted therapies for GB should persist, in which novel treatments based on natural compounds appear as a promising and attractive approach.

Medicinal plants are widely employed in traditional medicine to treat several illnesses, being a major source of bioactive compounds with high therapeutic value, which turn them attractive for ethnomedicine research (Blowman et al., 2018). These compounds are secondary metabolites produced by plants as a defense mechanism against predators and adverse environmental conditions, which explain their appealing biological activities (e.g., anti-inflammatory, antioxidant, antiproliferative, and antitumor) (Cragg and Pezzuto, 2016; Rayan et al., 2017; Howes, 2018). Based on this, the chemical structure of natural compounds grants them a unique mechanism of action against complex diseases such as cancer, which turn them into major players in the discovery of new anticancer drugs (Rayan et al., 2017; Howes, 2018). An example of bioactive natural compounds is terpenes, a class of secondary metabolites well-known for their described chemopreventive and chemotherapeutic activity (e.g., paclitaxel, an anticancer drug used in the clinic to treat ovarian and breast cancer) (Huang et al., 2012; Howes, 2018; Ansari and Akhtar, 2019; Yang et al., 2020a; Cheng et al., 2021).

Bearing this in mind, *Plectranthus* species are an example of plants rich in naturally occurring abietane diterpenes, the secondary metabolites responsible for the wide and attractive therapeutic bioactivities attributed to them. Moreover, Parvifloron D (ParvD), an abietane diterpene isolated from the plant *Plectranthus ecklonii* Benth. (*P. ecklonii*), is an example of a highly cytotoxic compound that has been studied as an anticancer agent against various cancer types (Santos-Rebelo et al., 2019; Saraiva et al., 2020; Śliwiński et al., 2020). Previous studies acknowledged different antitumor mechanisms of action of ParvD in pancreatic and breast cancers, either by inducing cell cycle arrest in G0/G1 phase (antiproliferative and cytostatic activity) or by promoting apoptosis and inhibiting cell migration and invasion (antimetastatic activity), respectively, showing the unthinkable therapeutic potential of this abietane diterpene (Santos-Rebelo et al., 2019; Saraiva et al., 2020).

In the current work, we aim to explore the antitumor potential of ParvD as a natural lead compound for future drug developments to treat GB. Moreover, we evaluated the antiproliferative/cytotoxic activity of ParvD in GB cell lines and unveiled its potential pro-apoptotic profile.

## Materials and methods

### Plant material

*P. ecklonii* whole plant samples were provided by the Faculty of Pharmacy of the University of Lisbon from seeds provided by the Kirstenbosch National Botanical Gardens (Kirstenbosch, South Africa). The voucher specimens (S/No.LISC) were deposited in the herbarium of the Tropical Research Institute in Lisbon (Burmistrova et al., 2015).

The plant was air dried (25°C) and stored in cardboard boxes protected from light and humidity to maintain stability. The plant name was confirmed with the repository http://www.worldfloraonline.org/ (Flora, 2022).

### Isolation of parvifloron D

**FIGURE 1:**
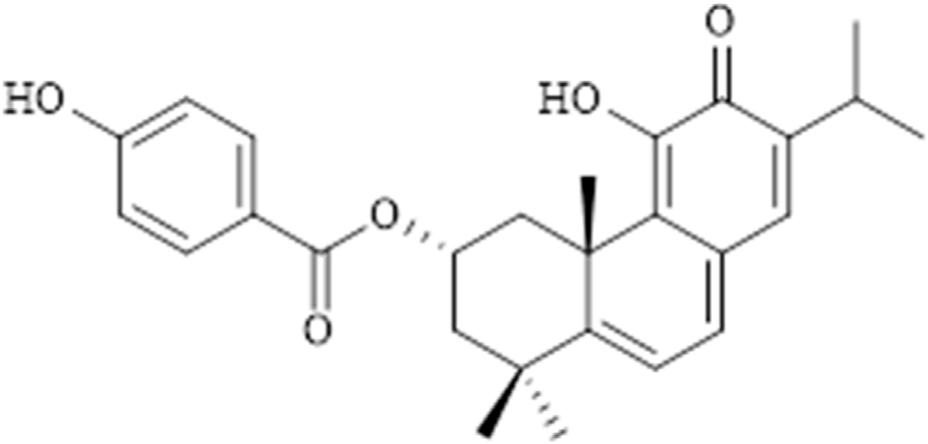
Chemical structure of parvifloron D.

*P. ecklonii* ultrasound-assisted extraction, using acetone as a solvent, was carried out as previously described (Santos-Rebelo et al., 2018). Afterward, the extract was fractionated through a Dry Flash Column Chromatography over silica gel, using mixtures *n*-hexane:ethyl acetate of increasing polarity monitored by TLC, allowing the obtention of fractions rich in ParvD. Finally, these fractions were repeatedly subjected to a Büchi Separacore^®^ automated system consisting of silica gel columns and as mobile phase mixtures of the increasing polarity of *n*-hexane:ethyl acetate and *n*-hexane:acetone, which allowed the isolation of pure ParvD (0.047 g). The chemical structure of ParvD (Figure 1) was elucidated in agreement with the data formerly published (Simões et al., 2010) and drawn using ChemDraw 21.0.0. software (Perkin-Elmer Informatics).

### Cell culture

U87 (brain-likely glioblastoma), A172 (glioblastoma), and H4 (neuroglioma) cell lines were kindly provided by Prof. Carla Vitorino (Faculty of Pharmacy, University of Coimbra), while U118 (astrocytoma/glioblastoma) and U373 (astrocytoma/glioblastoma) cell lines were kindly obtained by Prof. Maria Conceição Pedroso de Lima (Faculty of Sciences and Technology/Center for Neuroscience and Cell Biology, University of Coimbra). Cells were cultivated in Dulbecco’s Modified Eagle’s Medium-high glucose (DMEM-HG) (Biowest, Nuaillé, France) supplemented with 10% (v/v) of heat-inactivated fetal bovine serum (FBS) (Sigma, St. Louis, MO, United States) and 1% (v/v) of penicillin-streptomycin (Sigma, St. Louis, MO, United States), being maintained at 37°C and 5% CO_2_.

### Cell viability

U87, A172, U118, U373, and H4 cells (1 × 10^4^ cells/well) were seeded in 96-well plates, 24 h before treatment. Cells were treated with a range of ParvD concentrations (1, 4, 14, 58, 115, and 230 µM), with the vehicle control (DMSO), or with positive control (TMZ), and further incubated for 48 h. Cell viability was assessed through a modified Alamar blue assay (Magalhães et al., 2014). Briefly, a solution was prepared of DMEM-HG medium with 10% (v/v) of a resazurin salt dye stock solution (Sigma. St. Louis, MO, United States) at a 0.1 mg/ml concentration, which was further added to each well after 48 h treatment. After 4 h of incubation at 37°C and 5% CO_2_, the absorbance of the plate was read at 570 and 600 nm in a BioTeck (BioTek Instruments, Inc., Winooski, VT, United States). The absorbance results were obtained by the Gen5 program. Cell viability was then calculated in accordance with the following **equation**:

$$Cell\;viability\;\left(\%\right)=\frac{\left(A570-A600\right)\;of\;treated\;cells}{\left(A570-A600\right)\;of\;control\;cells\;}\times 100\%$$

Half-maximal inhibitory concentration (IC_50_) values were further calculated using GraphPad Prism Software v.7.04 (GraphPad Software, Inc.) (Beeby et al., 2021).

### Cell death assay

Assessment of cell death was achieved using the Annexin V (AV)/propidium iodide (PI) assay by flow cytometry. Twenty-four hours before treatment, U87, A172, U118, U373, and H4 cells (20 × 10^4^ cells/well) were seeded in 12-well plates. Cell medium was replaced and different concentrations of ParvD (1, 4, and 14 µM) were added to the cells. After 48 h treatment, cells were co-stained with AV-APC and PI according to the manufacturer’s protocol (Biolegend, San Diego, CA, United States). Cells were resuspended in binding buffer (100 µl) and incubated with AV-APC solution (5 µl) and PI solution (2 µl). Then, cells were diluted in binding buffer (400 µl). Data were acquired and treated as previously described (Magalhães et al., 2018).

### Cell cycle analysis

The Cell cycle was analyzed by flow cytometry using PI/RNase solution, according to the manufacturer’s protocol (Immunostep, Salamanca, Spain). U87, A172, U118, U373, and H4 cells (30 × 10^4^ cells/well) were seeded 24 h before treatment. Afterward, cells were treated with different ParvD concentrations (1, 4, and 14 µM). Cells were detached, fixed in 70% ethanol for 60 min at 4°C, washed twice with PBS, and then stained with 500 µl PI/RNase solution. Results were acquired using CellQuest software and analyzed to calculate the percentage of the cell population in each cell cycle phase.

### Mitochondrial membrane potential measurement

Mitochondrial membrane potential (ψmit) in U87, A172, U118, U373, and H4 cells treated with ParvD were measured using 5,5,6,6′-tetrachloro-1,1′,3,3′ tetraethylbenzimi-dazoylcarbocyanine iodide (JC-1) (Molecular Probes) as previously described (Gonçalves et al., 2013). Briefly, cells were treated with different ParvD concentrations (1, 4, and 14 µM) and further incubated for 48 h. After incubation, cells were washed twice with PBS, centrifuged at 3,450 rpm for 5 min, and incubated with JC-1 at a final concentration of 5 μg/ml for 15 min at 37°C in the dark. At the end of the incubation period, the cells were washed twice with PBS, resuspended in a total volume of 500 μl, and analyzed by flow cytometry.

### Quantitative real-time PCR

Expression levels of *B-cell lymphoma-2* (*Bcl2*), *Bcl-2 Associated X-protein* (*Bax*), *Bcl-2-like protein 1* (*BCL2L1*), *caspase 9*, *Phosphatase And Tensin Homolog* (*PTEN*), and *tumor protein p53* (*TP53*) mRNA were assessed by real-time PCR. U87, A172, U118, U373, and H4 cells (1 × 10^6^ cells/well) were seeded 24 h before transfection. Cells were treated with different ParvD concentrations (1 and 4 µM) and further incubated for 48 h. Total RNA was extracted using TripleXtractor solution (GRISP, Lisbon, Portugal) according to the manufacturer’s protocol. RNA was converted in cDNA through the Xpert cDNA Synthesis Supermix (GRISP, Lisbon, Portugal). cDNA was amplified by quantitative real-time PCR (qRT-PCR) using the primers in Table 1. Relative gene expression was determined by the 2^−ΔΔCt^ method and normalized to *Glyceraldehyde-3-Phosphate Dehydrogenase* (*GAPDH*), which was the endogenous reference, and relative to the untreated control cells.

**TABLE 1:** Sequence of the primers used in quantitative real-time PCR (qRT-PCR).

Gene	Forward (5′-3′)	Reverse (5′-3′)
*Bcl-2*	GAG​GAT​TGT​GGC​CTT​CTT​TGA​G	AGC​CTC​CGT​TAT​CCT​GGA​TC
*BCL2L1*	GCC​ACT​TAC​CTG​AAT​GAC​CAC​C	AAC​CAG​CGG​TTG​AAG​CGT​TCC​T
*Bax*	TCA​GGA​TGC​GTC​CAC​CAA​GAA​G	TGT​GTC​CAC​GGC​GGC​AAT​CAT​C
*Caspase 9*	GTT​TGA​GGA​CCT​TCG​ACC​AGC​T	CAA​CGT​ACC​AGG​AGC​CAC​TCT​T
*PTEN*	TGA​GTT​CCC​TCA​GCC​GTT​ACC​T	GAG​GTT​TCC​TCT​GGT​CCT​GGT​A
*TP53*	CAG​CAC​ATG​ACG​GAG​GTT​GT	TCA​TCC​AAA​TAC​TCC​ACA​CGC
*GAPDH*	GTC​TCC​TCT​GAC​TTC​AAC​AGC​G	ACC​ACC​CTG​TTG​CTG​TAG​CCA​A

### Statistical analysis

Data were analyzed using GraphPad Prism v.7.04. All experiments were performed in triplicate and acquired results were expressed as mean ± SD. Statistical analysis was performed by t-student test, one-way and two-way ANOVA, using the unpaired comparison and the multiple comparisons tests Tukey and Dunnett, respectively. A value of *p* < 0.05 was considered significant.

## Results

### Cell viability

**FIGURE 2:**
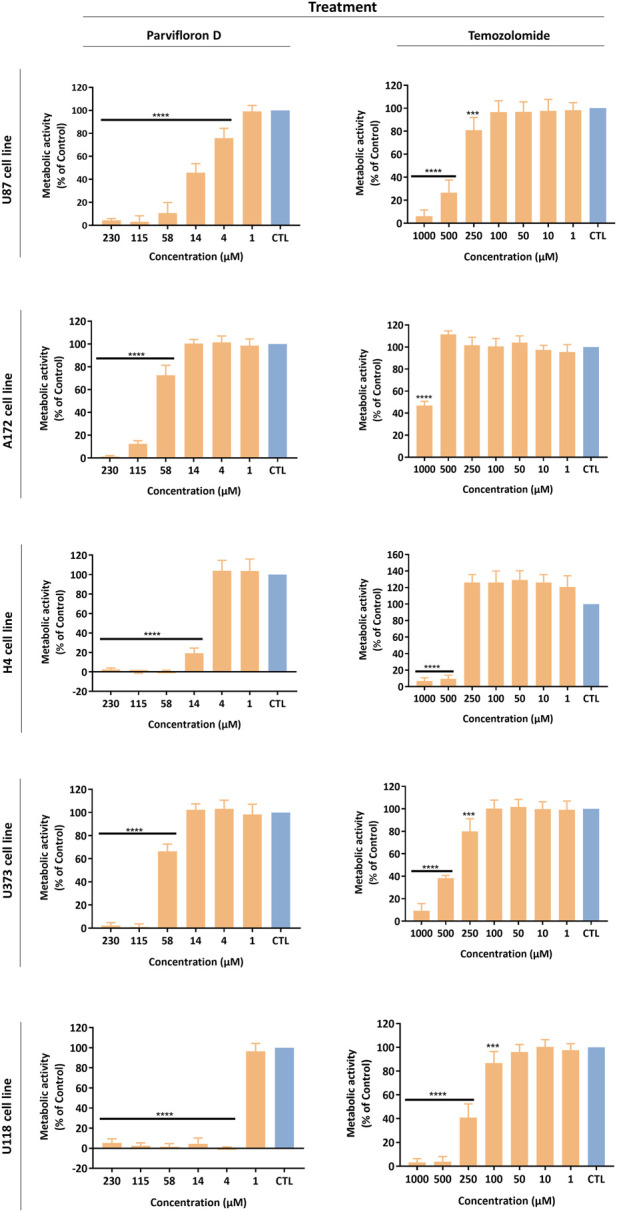
Antiproliferative/cytotoxic activity of Parvifloron D and temozolomide against U87, A172, H4, U373, and U118 cell lines. Cells were treated either with Parvifloron D or with temozolomide for 48 h and subsequently Alamar blue assay was performed. Cell viability was expressed as percentage of control (CTL) (untreated cells). Asterisks (****p < 0.0001) represent the values that significantly differ from the control. Data were presented as mean ± SD and are representative from at least three independent experiments.

The antiproliferative/cytotoxic potential of ParvD isolated from *P. ecklonii* was assessed and compared with the chemotherapeutic drug TMZ (first-line treatment for GB). The impact of ParvD and TMZ treatment on cell viability was evaluated in five glioma cell lines, comprising cells from primary and secondary GB to improve the *in vitro* model used for this tumor type (Figure 2). ParvD induced a significant decrease in the viability of GB cells (Figure 2). This cytotoxic effect was more pronounced if compared with TMZ treatment (Figure 2). Moreover, the cell metabolic activity was evidently more inhibited by ParvD and TMZ in U87, U118, and H4 cell lines (Figure 2). This was also supported by the half-inhibitory concentration (IC_50_) values (Table 2). Hence, U87, H4, and U118 cells were more sensitive to treatment than A172 and U373 cells. The ParvD doses needed to decrease the cell viability by 50% were much lower in all cell lines than the TMZ concentrations (Table 2). Furthermore, the IC_50_ values to induce cell death in U87 (11.28 µM), H4 (12.47 µM), and U118 (1.22 µM) cells were 33, 36, and 175 times, respectively, less than the corresponding TMZ concentrations (Table 2).

**TABLE 2:** Half-maximal inhibitory concentration (IC_50_) values for parvifloron D and temozolomide in a panel of GB cell lines.

Compound	IC_50_ (µM)				
	U87 cell line	A172 cell line	H4 cell line	U373 cell line	U118 cell line
*Parvifloron D*	11.28	72.01	12.47	59.68	1.22
*Temozolomide*	371.21	948.39	444.91	409.01	213.49

### Cell death assessment

**FIGURE 3:**
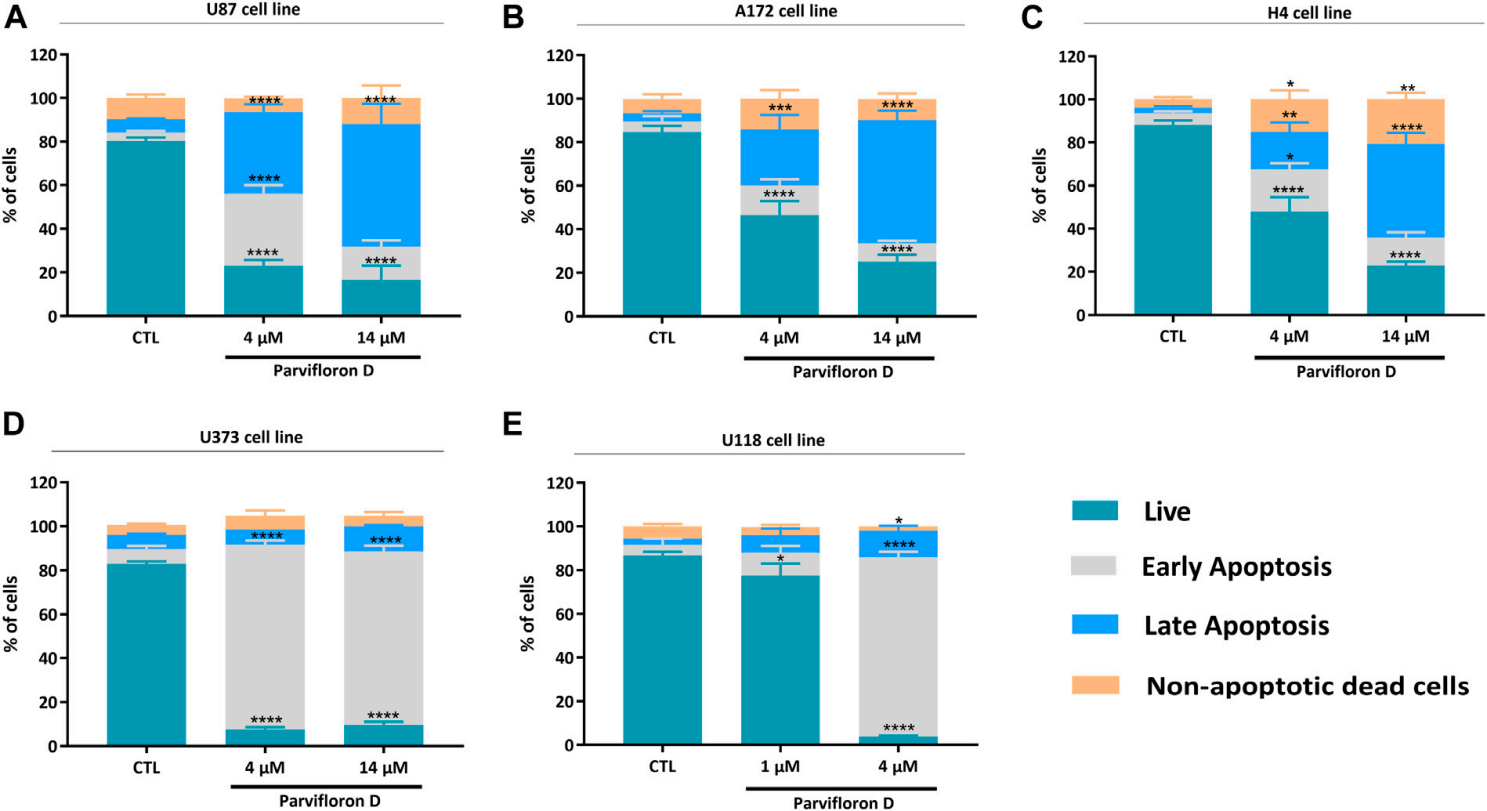
Assessment of cell death profile in U87 **(A)**, A172 **(B)**, H4 **(C)**, U373 **(D)**, and U118 **(E)** cell lines by flow cytometry. Cells were treated with parvifloron D and further incubated for 48 h. After incubation, cells were co-stained with Annexin V and PI and the percentage of non-apoptotic cells or cells in apoptosis was determined. Asterisks (*p < 0.05, **p < 0.01, ***p < 0.001, and ****p < 0.0001) represent the values that significantly differ from the values obtained for the control group (untreated cells). These results are representative of at least five independent experiments and data expressed as mean ± SD.

The results obtained from the cell viability assays showed the therapeutic potential of ParvD to surpass cellular resistance associated with TMZ. As a next step, we assessed the cell death mechanism using AV/PI staining, which enables to differentiate live cells from cells in apoptosis or necrosis based on the permeability and integrity of the plasma membrane. All cell lines were treated with concentrations of 4 and 14 µM ParvD, except for U118 cells that were treated with 1 and 4 μM, for 48 h and further analyzed by flow cytometry (Figure 3; Supplementary Figure S1). As expected, ParvD treatment significantly decreased the percentage of viable cells relative to the untreated control cells (*p < 0.05; ****p < 0.0001). Moreover, this decrease of live cells was more pronounced in U373 cells for both tested concentrations (****p < 0.0001) (reduction of viable cells >80%) and in U118 cells (decrease of viability by 90%) after treatment with 4 µM (****p < 0.0001) (Figures 3D,E). These results were accompanied by an increase in the percentage of cells in early and late apoptosis/necrosis (Figure 3). Also, higher percentages of cells in early apoptosis were observed in astrocytoma/GB cells (Figures 3D,E), while an increasing number of cells were found in late apoptosis/necrosis in GB (Figures 3A,B) and neuroglioma (Figure 3C) cell lines.

**FIGURE 4:**
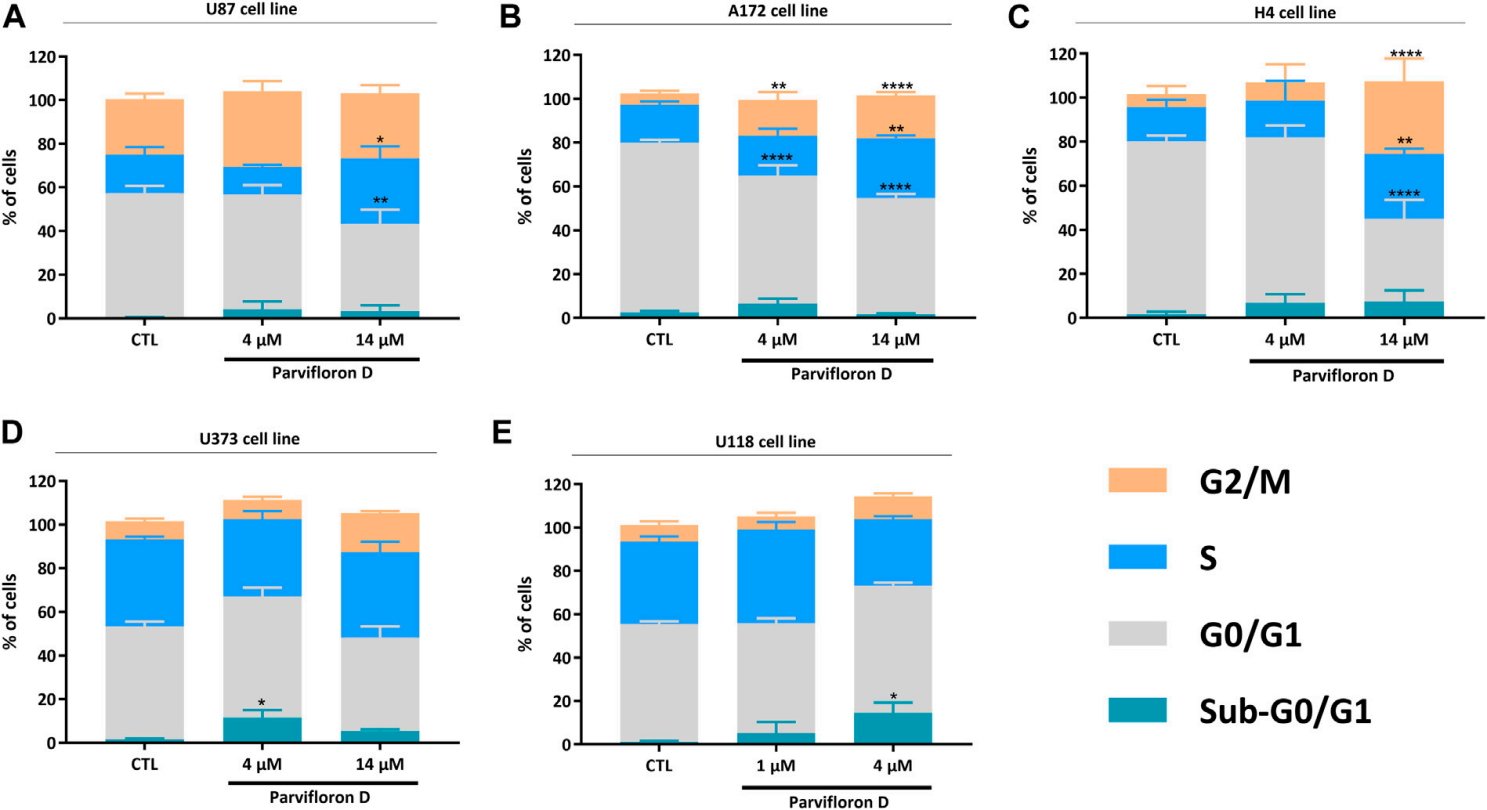
Evaluation of parvifloron D treatment on cell cycle progression in U87 **(A)**, A172 **(B)**, H4 **(C)**, U373 **(D)**, and U118 **(E)** cell lines. Cells were treated with parvifloron D for 48 h. After incubation, cells were stained with PI/RNase and the cell cycle was assessed by flow cytometry. The proportion of cells in subG0/G1, G0/G1, S, and G2/M cell cycle phases was expressed as a percentage of the total cell population. Asterisks (*p < 0.05, **p < 0.01, and ****p < 0.0001) represent the values that significantly differ from the values obtained for the control condition (untreated cells). Data are representative of at least five independent experiments and are expressed as mean ± SD.

Considering the increase in the number of cells in early and late apoptosis/necrosis, we explored the probable mechanism responsible for this outcome. The effect of ParvD treatment on cell cycle regulation was evaluated using PI/RNase staining and flow cytometry (Figure 4). Quantification of DNA content permitted to show an arrest in S and G2/M phase in U87, A172, and H4 cells (Figures 4A–C) but not in U373 and U118 cells (Figures 4D,E), if compared with the control condition. An increase of the subG0/G1 fraction was observed for ParvD treatment in all cell lines (Figure 4), being more significant in U373 and U118 cells (*p < 0.05) (Figures 4D,E). The evident increase of subG0/G1 in U373 and U118 cells was expected, which confirms the higher percentage of cells in early apoptosis (Figures 3D,E). Also, higher subG0/G1 fractions were found in U87, A172, and H4 cells (Figures 4A–C), indicating that ParvD promoted apoptosis rather than necrosis (Figures 3A–C).

### Mitochondrial membrane potential

Next, we assessed the mitochondrial involvement in ParvD-induced cell death using JC-1 and flow cytometry. JC-1 is a lipophilic and cationic dye that accumulates into the mitochondria and forms aggregates in healthy cells, while in apoptotic cells, the mitochondrial membrane potential gets lost and JC-1 cannot accumulate inside the mitochondria, maintaining its monomeric form in the cytosol. Therefore, the higher monomer/aggregate (M/A) ratio is associated with a decrease in the mitochondrial membrane potential and, subsequently, related to an increase of cells in apoptosis.

**FIGURE 5:**
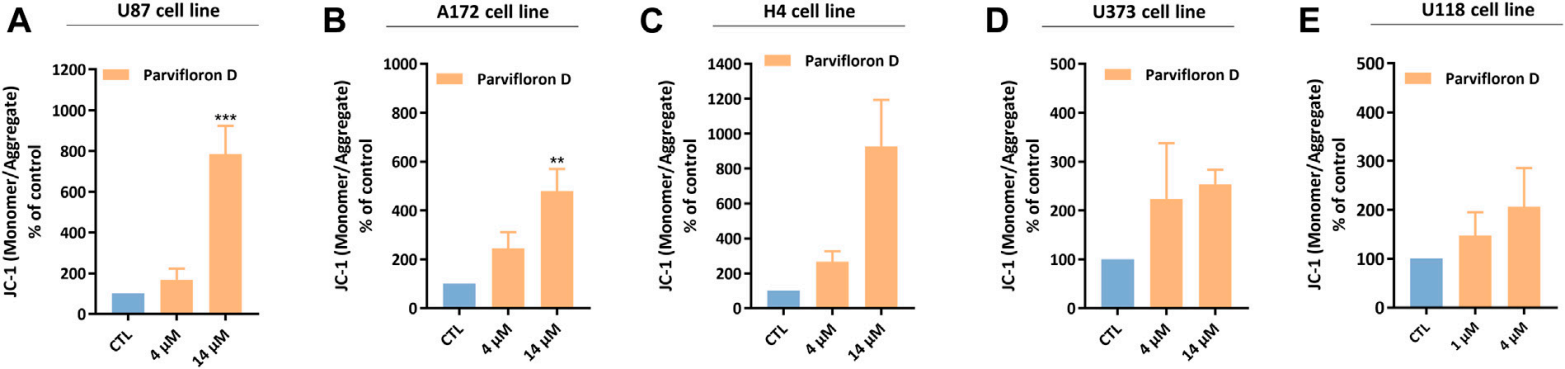
Analysis of the mitochondrial membrane potential in U87 **(A)**, A172 **(B)**, H4 **(C)**, U373 **(D)**, and U118 **(E)** cell lines after treatment with parvifloron D for 48 h. The monomeric or aggregate form of JC-1 probe depends on the mitochondrial membrane potential. An increase in the monomer/aggregate (M/A) ratio indicates a decrease in the mitochondrial membrane potential. Results are expressed as M/A ratio of JC-1, which was calculated as the fraction of MFI observed for each molecule. Asterisks (**p < 0.01 and ***p < 0.001) represent the values that significantly differ from the control (untreated cells). Data were presented as mean ± SD and are representative of at least three independent experiments.

An increase in the monomer/aggregate (M/A) ratio was observed for the conditions treated with ParvD compared to viable cells (control condition) in the five cell lines (Figure 5). Moreover, an enhancement of M/A ratio, was more pronounced in GB (Figures 5A,B) and neuroglioma (Figure 5C) cells after treatment with 4 µM ParvD, while astrocytoma/GB cells showed the lowest M/A ratios (Figures 5D,E). However, a similar profile was observed in all cell lines with a proportional increase of M/A ratio upon increasing ParvD concentrations (dose-dependent effect) (Figure 5). These results further support the hypothesis that cell death was induced by apoptosis after treatment with ParvD for 48 h.

### Assessment of mRNA levels of genes associated with pro-apoptotic mechanisms

**FIGURE 6:**
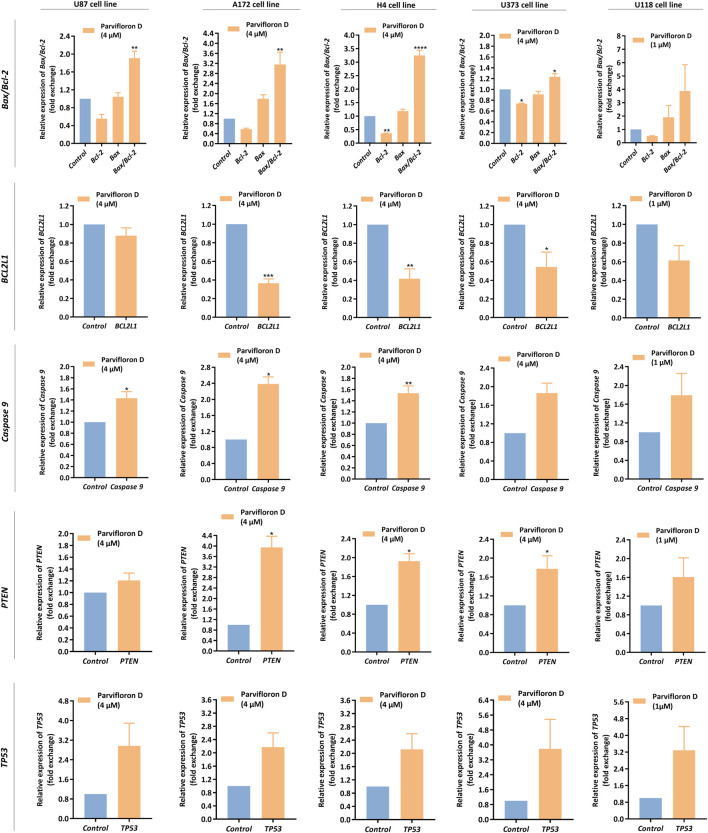
Assessment of *Bax*, *Bcl-*2, *BCL2L1*, *Caspase 9*, *PTEN*, and *TP53* mRNA levels in U87, A172, H4, U373, and U118 cells, after parvifloron D treatment for 48 h. After incubation, the relative expression of *Bax*, *Bcl-2*, *BCL2L1*, *Caspase 9*, *PTEN*, and *TP53* was assessed by qRT-PCR and the results normalized to *GAPDH* expression. Asterisks (*p < 0.05, **p < 0.01, ***p < 0.001, and ****p < 0.0001) show the significant difference between the values obtained for treated cells relative to the control group (untreated cells). Data are presented as mean ± SD and it is representative of at least three independent experiments.

In the aforementioned data, ParvD induced cell death by apoptosis in GB cells. Thus, we assessed the mRNA levels of genes encoding proteins involved in the modulation of pro-apoptotic mechanisms. We measured the *Bcl-2*, *BCL2L1*, *Bax, Caspase 9*, *PTEN,* and *TP53* mRNA levels in GB cells, after treatment with ParvD for 48 h (Figure 6).

We observed a pronounced decrease in the *Bcl-2* and *BCL2L1* mRNA expression levels and a slight increase in *Bax* mRNA expression levels relative to the control group (untreated cells). Moreover, the *Bax/Bcl-2* ratio significantly increased, mainly in U87, A172, and H4 cells (Figure 6), which may indicate that ParvD treatment downregulated anti-apoptotic genes (*Bcl-2* and *BCL2L1*) and upregulated pro-apoptotic genes (*Bax*), subsequently activating the intrinsic mitochondria-dependent apoptotic pathway.

Based on the increase of *Bax/Bcl-2* mRNA expression ratio, we also measured the *Caspase 9* mRNA levels. Once Bcl-2 is suppressed, Bax is activated, and cytochrome C is released from the mitochondria to the cytoplasm, causing activation of caspase-9 and -3. Indeed, we observed that ParvD treatment caused a substantial upregulation of *caspase 9* mRNA levels in all cell lines (Figure 6), suggesting that cell death occurred by apoptosis *via* the caspase-dependent mitochondrial pathway.

Additionally, we also quantified the mRNA levels of the tumor suppressor genes *PTEN* and *TP53*. *PTEN is* a negative regulator of phosphatidylinositol 3-kinase (PI3K)/protein kinase B (Akt) signaling pathway, while *TP53* encodes p53 protein, a transcriptional regulator known as the “guardian of the genome,” that acts upon stress signals to prevent DNA damage and induce apoptosis and cell cycle arrest. Both tumor suppressor genes were downregulated in GB cells, but ParvD treatment increased the *PTEN* and *TP53* mRNA levels in GB cells (Figure 6).

The qRT-PCR data are in line with the results obtained along this work (Figures 2–5), suggesting once more that ParvD induced mitochondrial-dependent cell death.

## Discussion

Natural products are an important source of biologically active molecules with high therapeutic value, which stirred the attention of ethnomedicines (Rodrigues et al., 2016). Since ancient times, the use of natural products in traditional medicine has been entrenched in all cultures, representing a major slice of the primary mode of healthcare in developing countries (WHO, 2011; Magalhães et al., 2021). In countries with a high human development index, natural products and natural products-based drugs are also used as an adjuvant and complementary therapeutics for various illnesses (Apaya et al., 2016). Presently, over 60% of the chemotherapeutic drugs approved by the Food and Drug Administration (FDA) and by the European Medicines Agency (EMA) derive from natural sources or are inspired by the chemical structure of natural compounds (e.g., the diterpene paclitaxel and the alkaloids vinblastine and vincristine, which are used to treat ovarian and breast cancer and leukemia, respectively) (Cragg and Pezzuto, 2016; Rayan et al., 2017; Howes, 2018). Therefore, the unique structure and mechanism of action of natural compounds offer an invaluable variety of potential new therapeutic drugs to improve cancer treatment (Khazir et al., 2014; Cragg and Pezzuto, 2016; Rodrigues et al., 2016; Rayan et al., 2017). Therefore, the design of novel therapies based on natural drug leads emerges as a viable and worthwhile approach to treat GB, a highly malignant glioma of the CNS that still act as a massive therapeutic challenge, mainly due to its poor prognosis (Louis et al., 2021; Magalhães et al., 2021). Terpenes, the second biggest class of secondary metabolites in plants, are feasible examples of natural compounds with enhanced therapeutic value, including anticancer activity, which are already recognized by different health-based industries, namely the cosmetic, food and pharmaceutical industry, due to their compelling bioactivities (Wang et al., 2005; Huang et al., 2012; Yang et al., 2020b; Proshkina et al., 2020; Gutiérrez-del-Río et al., 2021).

Herein, during this work, the antitumoral potential of ParvD, an abietane diterpene isolated from *P. ecklonii*, was explored as a potential natural drug lead for future therapeutic strategies against GB. Thereby, the chemotherapeutic activity of ParvD was assessed in a panel of five glioma cell lines, as an attempt to cover the heterogeneity of GB, which can either arise *de novo* or following low-grade gliomas.

Treatment with ParvD caused a significant decrease in the viability and proliferative capability of GB cells, exhibiting a more pronounced cytotoxic/antiproliferative effect if compared with TMZ treatment (first-line treatment) (Figure 2). Moreover, for all cell lines, the ParvD concentrations required to reduce the cell viability by 50% were considerably lower than the ones of TMZ needed to induce similar effects (Table 2). The strongest impact of ParvD was observed for U118, U87, and H4 cells with IC_50_ values of 1.22, 11.28, and 12.47 µM, respectively. These values were approximately 175, 33, and 36 times lower than the TMZ half-inhibitory concentrations for these cell lines (371.21, 444.91, and 213.49 µM for U87, H4, and U118 cells, respectively) (Table 2). Relatively to the results for A172 and U373 cell lines, we obtained higher IC_50_ values for ParvD (72.01 and 59.68 µM, respectively), compared with the data for U118, U87, and H4 cells. However, ParvD still revealed a better antiproliferative/cytotoxic activity, requiring concentrations around 13 to 7 times lower than the ones of TMZ necessary to inhibit proliferation by 50% in A172 and U373 cells (948.39 and 409.01 µM) (Table 2). This preliminary analysis concluded that ParvD had a strong cytotoxic/antiproliferative activity against GB cells, which complies with the cytotoxic profile obtained for this diterpene in cell lines from different tumors (Śliwiński et al., 2020). Therefore, we performed an in-depth investigation to unveil whether the cell death mechanism induced by ParvD was cytostatic (only affects cell proliferation) or cytotoxic (increases cell death).

Thus, assessment of cell death after ParvD treatment showed a cytotoxic effect in GB cells sustained by the significant increase of cells in early and late apoptosis (Figure 3). Furthermore, an enhancement of cells in early apoptosis was more noticeable in U373 and U118 cells (Figures 3D,E), which was also supported by the increasing percentage of cells in the subG0/G1 phase (Figures 4D,E) (indicative of DNA fragmentation and related to an increase of apoptotic cells) (Plesca et al., 2008). Meanwhile, U87, A172, and H4 cells contained a higher percentage of cells in early apoptosis but also a substantial augment of cells in late apoptosis/necrosis (Figures 3A–C). Through cell cycle analysis, we observed an increase in cell population in subG0/G1, corroborating that ParvD provoked cell death by apoptosis (Figures 4A–C). ParvD also caused a dose-dependent block in S and G2/M phases, accompanied by a decrease in cell population in G0/G1 phase in glioma cells (Figures 4A–C). ParvD probably induces DNA damage and cell cycle arrest by downregulation of the cyclin-dependent kinase1 (cdk1)/cyclin B1 complex through activation of p53 and a transcriptionally increase of p21 expression levels (Shangguan et al., 2014; Wang et al., 2016; Fallahian et al., 2017; Doan et al., 2019). Furthermore, upregulation of *PTEN* and *TP53* mRNA levels in glioma cells was observed following treatment with ParvD compared to untreated control (Figure 6), perhaps indicating an inhibition of the PI3K/Akt signaling pathway through upregulation of *PTEN* mRNA levels and subsequent inhibition of mouse double minute 2 (MDM2) activity and restoration of p53 levels (Pearson and Regad, 2017).

As previously discussed, our results suggested that ParvD induced cell cycle arrest and apoptosis. However, it should also be explored whether cell death occurred *via* intrinsic and/or extrinsic apoptotic pathways. Apoptosis is a type of programmed cell death that involves highly conserved and controlled mechanisms responsible for ordering cells to commit suicide and remove damaged or harmful cells, being an essential process to maintain tissue homeostasis (Elmore, 2007; Shangguan et al., 2014; Mishra et al., 2018). Thus, apoptosis can be triggered either *via* activation of the extrinsic pathway, which is the cell death receptor-dependent, or by activation of the intrinsic pathway, also known as the mitochondria-dependent pathway (regulated *via* Bcl-2 family) (Campbell and Tait, 2018; Bedoui et al., 2020; Fanfone et al., 2020). Although, a completely different scenario occurs within cancer cells, with a dysregulation of the upstream apoptotic signaling processes, revealing an inability to trigger apoptosis in response to cellular stresses, like DNA damage or upregulation of oncogenes (Mishra et al., 2018; Bedoui et al., 2020; Fanfone et al., 2020). After GB cells were treated with ParvD, the expression of pro-apoptotic genes, related to the intrinsic pathway, and the impact of mitochondria on cell death were analyzed. ParvD treatment induced a dose-dependent decrease in the mitochondrial membrane potential in GB cells (Figure 5), which is accompanied by apoptosis induction (Gonçalves et al., 2013). In other words, a decrease in the mitochondrial membrane potential enhances the permeabilization of the mitochondrial outer membrane, leading to the release of cytochrome c into the cytosol, where it activates caspase-9 and, subsequently, caspase-3 and -7 (Fanfone et al., 2020). The mitochondria-dependent pathway is regulated by the Bcl-2 protein family, which includes anti-apoptotic (e.g., Bcl-2, Bcl-xl) and pro-apoptotic (e.g., Bax) proteins that are overexpressed and inhibited in GB, respectively (Campbell and Tait, 2018; Fanfone et al., 2020). ParvD induced a decrease in the *Bcl-2* and *BCL2L1* mRNA expression and an increase on the *Bax/Bcl-2* mRNA ratio (Figure 6). Moreover, increased *caspase 9* mRNA levels were also observed after ParvD treatment in GB cells (Figure 6).

Overall, the results in this study showed that ParvD has a strong cytotoxic effect in GB cells, inducing apoptosis *via* the mitochondria-dependent pathway, presumably through modulation of *Bcl-2* and *Bax* mRNA expression with activation of the caspase cascade. Further studies will be addressed in a GB-induced animal model to confirm the therapeutic effect of ParvD.

## Conclusion

The concentrations of ParvD required to reduce cell viability by 50% were substantially lower than those of TMZ (first-line treatment) required to promote similar effects. Moreover, treatment with ParvD induced cell death by apoptosis, which was supported by an increase of cell populations in the subG0/G1 phase and a decrease of the mitochondrial membrane potential as well as by the upregulation of pro-apoptotic and tumor suppressor genes. Therefore, ParvD could serve as a promising lead compound to overcome the limitations associated with the conventional treatment (e.g., multidrug resistance associated with TMZ). Further studies will be addressed to deepen our understanding of the antitumoral mechanism of action of ParvD.

## Data Availability

The original contributions presented in the study are included in the article/Supplementary Material, further inquiries can be directed to the corresponding authors.
