# Supplementary material for: Parvifloron D-based potential therapy for glioblastoma: Inducing apoptosis via the mitochondria dependent pathway
Source: Front Pharmacol. 2022 Oct 12;13:1006832. doi: 10.3389/fphar.2022.1006832 (PMC9605735; doi:10.3389/fphar.2022.1006832)
Supplement: Supplementary file 1 [file DataSheet1.docx]

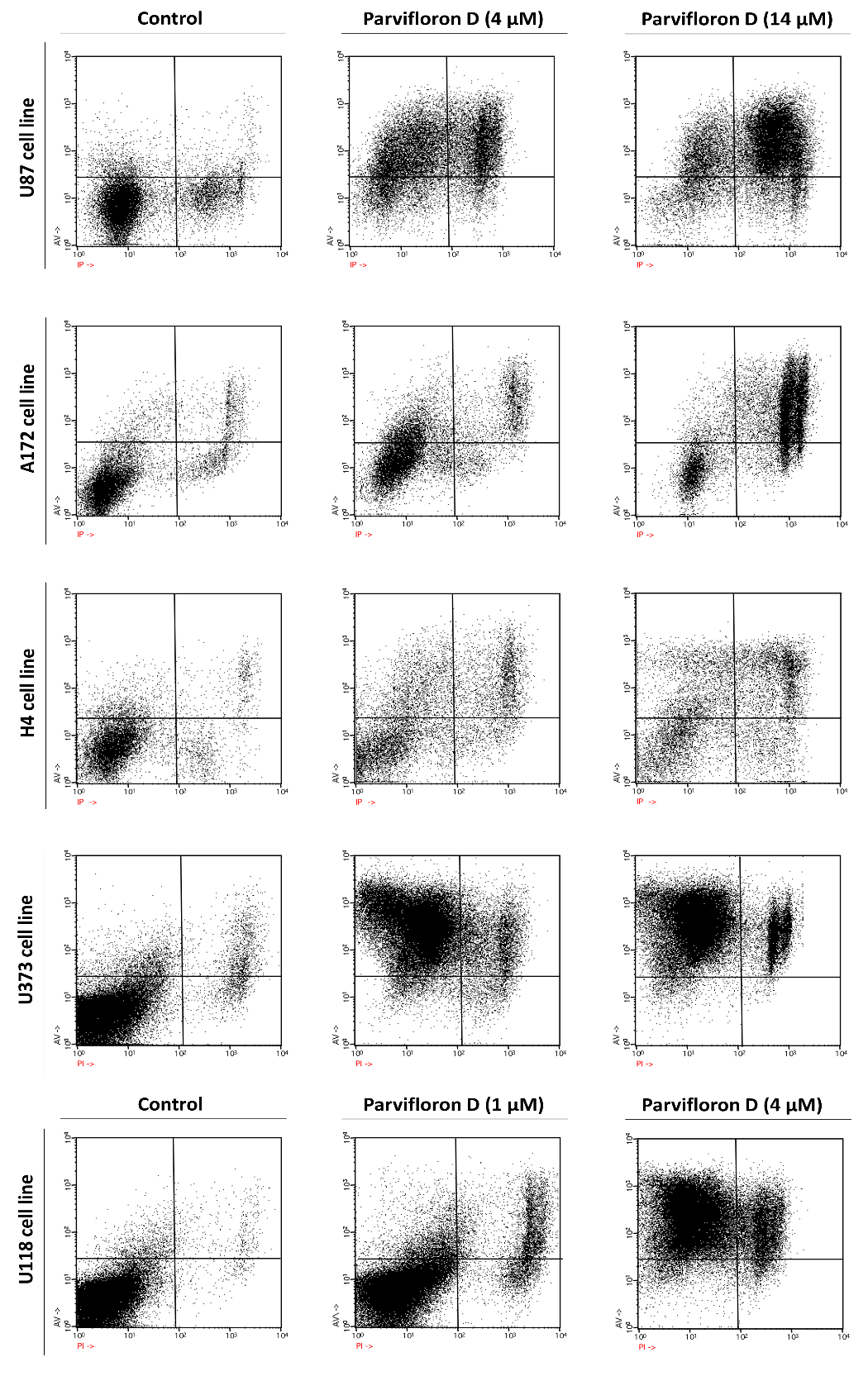


**Supplementary Figure 1.** Histograms representing cell death profile in U87, A172, H4, U373, and U118 cell lines. Cells were treated with parvifloron D and further incubated for 48 hours. After incubation, cells were co-stained with Annexin V and PI and data were analyzed by flow cytometry. **Note:** The lower left square represents live cells, the upper left square shows early apoptotic cells, the upper right square represents late apoptotic cells and the lower right square shows non-apoptotic cells.
